# Supplementary material for: Machine Learning of Temperature‐Dependent Chemical Kinetics Using Parallel Droplet Microreactors
Source: Adv Sci (Weinh). 2026 Aug 24;13(63):e77040. doi: 10.1002/advs.77040 (PMC13502267; doi:10.1002/advs.77040)
Supplement: Supplementary file 1 [file ADVS-13-e77040-s001.pdf]

Supporting Information:
Machine Learning of Temperature-dependent Chemical Kinetics Using Parallel Droplet Microreactors

Mamoru Saita, Yutaka Hori*

Department of Applied Physics and Physico-Informatics,
Faculty of Science and Technology, Keio University, Japan
{mamosaita64, yhori}@keio.jp

Supplementary Note 1: Immobilization of droplets

The image of the entire chamber at the beginning of the experiment ($t = 0$ min), corresponding to Fig. 2B, is shown in Fig. S1. The colored lines in the figure represent the trajectories of the droplet centers tracked over 180 min. Droplets without the trajectories correspond to those that could not be continuously tracked during the time course and were therefore excluded from the analysis. Figure S1 illustrates that the displacement of most detected droplets remains within approximately one droplet radius (100 µm) throughout the observation window (180 min), indicating stable immobilization of the droplets.

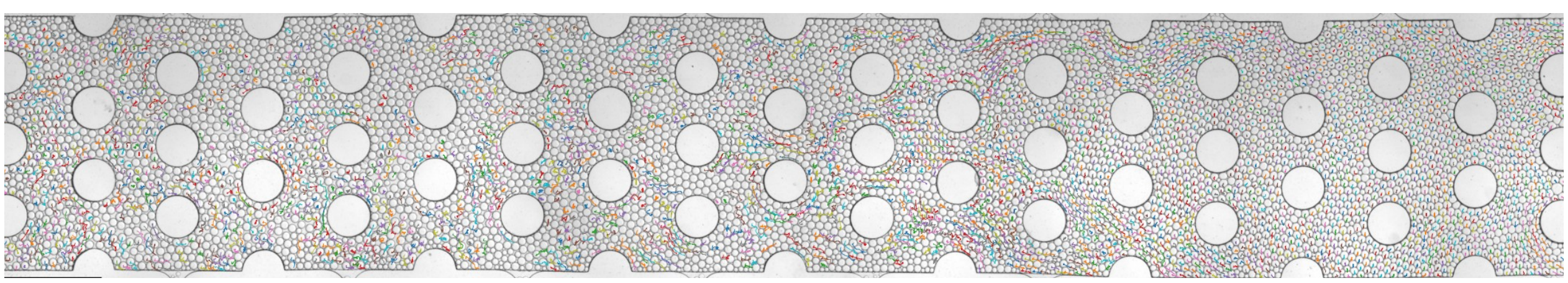

Figure S1: Trajectory analysis of droplets immobilized under a temperature gradient. Bright-field image of the entire chamber at 0 min is overlaid with the trajectories of droplets tracked for 180 min. Scale bar represents 1 mm.

Supplementary Note 2: Calibration of DNA thermometer

The calibration curve of the DNA thermometer in Fig. 2C was obtained by fitting the averaged fluorescence intensities of droplets measured at each temperature to a sigmoidal function using nonlinear least-squares optimization. Curve fitting was performed with the `curve_fit` function of the SciPy library [1] in Python, which employs the Levenberg–Marquardt (LM) algorithm for unconstrained minimization.

The fluorescence intensity F of the DNA thermometer as a function of temperature T was modeled as

$$F(T) = \frac{a}{1 + b\exp\left(c(T + d)\right)} + e,$$

where a, b, c, d, and e are fitting parameters. The fitted parameters are summarized in Table S1.

Table S1: Fitted parameters of the DNA thermometer calibration curve obtained by nonlinear least-squares fitting.

Parameter	Symbol	Fitted value
Amplitude	a	7659
Scaling factor	b	3.045
Slope coefficient	c	-0.1950
Temperature shift	d	-35.39
Offset	e	544.0

Precision of temperature readout

The precision of the temperature readout was further evaluated at the single-droplet level by inferring the temperature of each droplet from its fluorescence intensity using the calibration curve in Fig. 2C (Fig. S2A). Fig. S2A shows that, from 30 to approximately 48 °C, the interquartile ranges of the inferred temperatures were separated between adjacent 2°C temperature conditions. This indicates sufficient temperature differences of approximately 2°C can be reliably resolved over the temperature range used for the kinetic analysis in this study.

Temporal response of DNA thermometer The temporal response of the DNA thermometer was evaluated using a real-time PCR instrument (AriaMx, Agilent) under different temperature ramp rates. For each measurement, 20 µL of 2.0 µM DNA thermometer solution was loaded into four sample wells, and the fluorescence intensity was recorded during both heating and cooling ramps. The fluorescence–temperature trajectories obtained at ramp rates of approximately 10 s/°C or slower were nearly identical, whereas a faster ramp rate of 5 s/°C showed a noticeable deviation, indicating that the DNA thermometer can follow temperature changes at least on this timescale (Fig. S2B). It should be noted that, in the dynamic temperature gradient experiment in Fig. 4, the measured temperature trajectories varied on the order of approximately 30s/°C, which is slower than the response limit described above. This indicates that the response speed of the DNA thermometer was sufficient in this experiment.

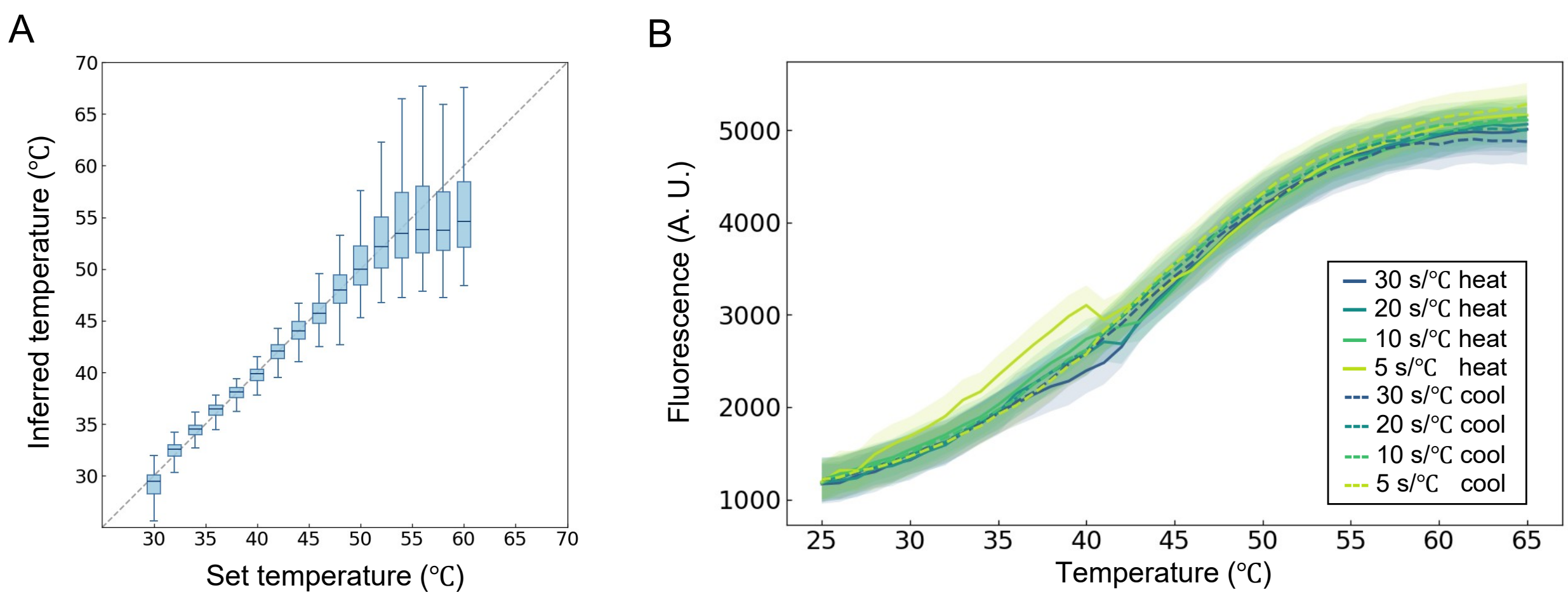

Figure S2: Precision of the temperature readout and temporal response of the DNA thermometer.(A) Distribution of droplet temperatures inferred from DNA thermometer fluorescence using the calibration curve in Fig. 2C. Boxes represent the interquartile range, center lines indicate the median, and whiskers represent the farthest data point within 1.5 times the IQR of the upper and lower quartiles. (B) Fluorescence–temperature trajectories of the DNA thermometer measured using a qPCR instrument under different temperature ramp rates. Solid and dashed lines correspond to heating (up) and cooling (down) ramps, respectively. Shaded bands indicate the standard deviation across four sample wells. The trajectories at approximately 10 s/°C or slower were nearly identical, whereas the faster 5 s/°C trajectory deviated, indicating that the DNA thermometer can follow temperature changes at least on the order of 10 s/°C.

Supplementary Note 3: Neural ODE model for stationary temperature gradient

Prediction performance under stationary temperature gradient

Figure S3 shows the Neural ODE predictions plotted against all test data under the stationary temperature gradient, corresponding to Fig. 3E in the main text, where only representative time points are displayed. The predicted fluorescence intensities showed a strong linear correlation with the experimental values, indicating that the Neural ODE model successfully reproduced the enzymatic reaction kinetics across the stationary temperature conditions.

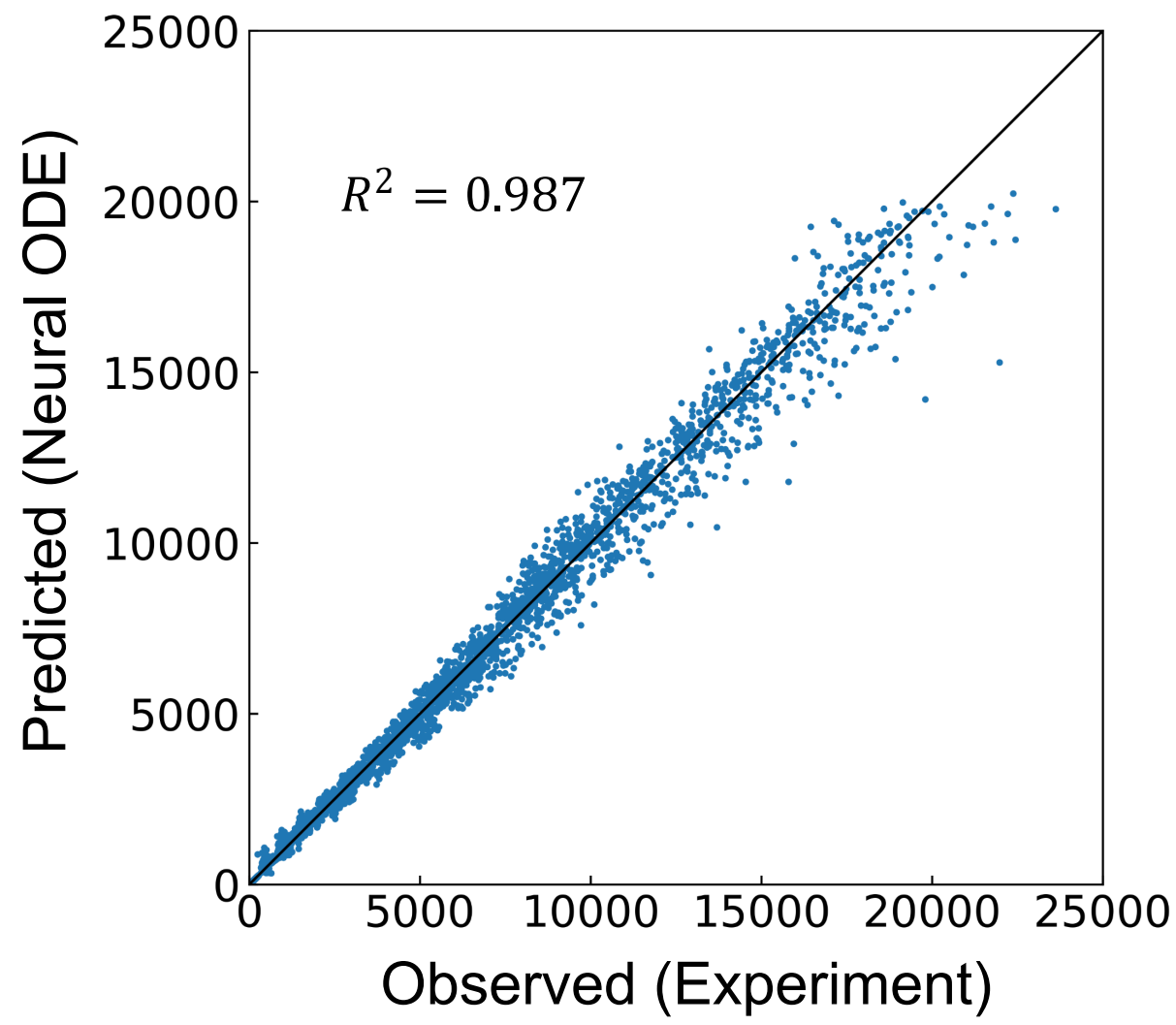

Figure S3: Neural ODE predictions plotted against all test data under the stationary temperature gradient.

Sensitivity of neural network architecture

To evaluate the robustness of the selected neural network architecture, we performed an architecture sensitivity analysis by varying the number of hidden layers and the number of neurons per hidden layer. Specifically, we tested networks with 1, 2, and 3 hidden layers and 32, 64, and 128 neurons per hidden layer. The resulting R^2 scores are summarized in Table S2.

For the models with one hidden layer, the predictive performance depended strongly on the number of neurons. Models with 32 and 64 neurons showed poor predictive accuracy, whereas increasing the number of neurons to 128 substantially improved the performance. In contrast, models with two or more hidden layers achieved consistently high predictive accuracy, with R^2 values of approximately 0.986–0.987. The selected architecture used for Fig. 3, consisting of two hidden layers with 64 neurons per hidden layer, achieved $R^2 = 0.987$, which was comparable to the best-performing architectures while maintaining a compact model size. These results indicate that the stationary temperature gradient dataset can be accurately modeled once sufficient model capacity is provided, and that the prediction is not highly sensitive to the exact number of neurons and hidden layers.

Table S2: Architecture sensitivity analysis of the Neural ODE model under the stationary temperature gradient. The selected architecture used for in Fig. 3 is shown in bold.

Number of hidden layers	Number of neurons	R^2
1	32	-0.0301
1	64	0.0275
1	128	0.986
2	32	0.987
**2**	**64**	**0.987**
2	128	0.987
3	32	0.986
3	64	0.986
3	128	0.986

Sensitivity analysis of data trimming fraction

Droplets located near the lateral edges of the observation chamber consistently exhibited lower fluorescence intensities than those in the central region (Fig. S4). Therefore, we excluded the outermost 5% on each lateral side from subsequent analyses. To assess the effect of the lateral-edge trimming fraction on the predictive performance, we performed a preprocessing sensitivity analysis under the stationary temperature gradient. For this purpose, datasets were generated for different trimming fraction from 0% to 10%, and an independent Neural ODE model was trained for each dataset using the same model architecture and hyperparameters. Since the number of available samples differed depending on the trimming fraction, the models were evaluated on the same common test set to ensure a fair comparison of predictive performance. The common test set consisted of 222 samples, corresponding to 2664 fluorescence data points. The numbers of training and validation samples for each trimming threshold are summarized together with the prediction performance in Table S3. The predictive performance remained the same across the tested trimming fractions. These results indicate that the prediction performance of the Neural ODE model was robust to the choice of the lateral-edge trimming fractions. This is partly because droplets whose DNA-thermometer fluorescence exceeded the range of the calibration curve (Fig. 2C) could not be assigned a temperature and had already been excluded as missing values prior to the lateral-edge trimming, independently of the trimming fraction.

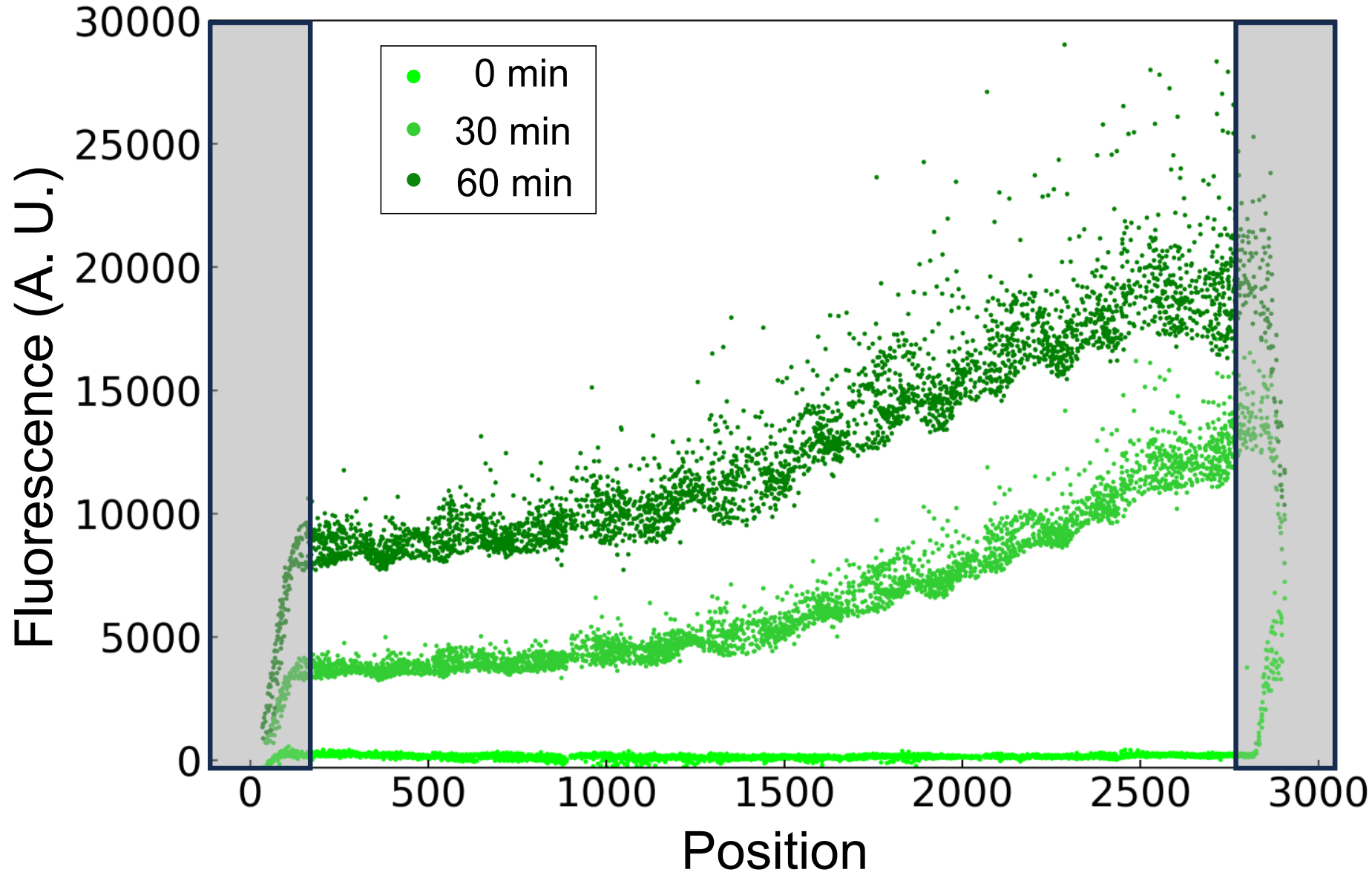

Figure S4: Spatial profiles of fluorescence intensities under the stationary temperature gradient. The gray hatched regions correspond to the excluded data from the analysis (5% on each lateral side).

Table S3: Sensitivity analysis of the Neural ODE model to the trimming fraction under the stationary temperature gradient. R^2 stands for the coefficient of determination between the predicted and measured fluorescence intensities on the test set.

Trimming fraction	R^2
0%	0.986
2.5%	0.986
5%	0.986
7.5%	0.986
10%	0.986

Supplementary Note 4: Ordinary Differential Equation (ODE) model

To benchmark the data-driven Neural ODE model against a conventional physics based model, we constructed an ODE model of the enzymatic reaction based on physics laws. The model parameters were then optimized to fit the experimental data taken under the stationary temperature gradient (Fig. 3).

The substrate and product concentrations were modeled based on the Michaelis–Menten kinetics by

$$\frac{dS(t)}{dt} = -\frac{1}{B}\frac{V_{\max}(T)\,S(t)}{K_m + S(t)}, \tag{1}$$

$$\frac{dP(t)}{dt} = \frac{V_{\max}(T)\,S(t)}{K_m + S(t)}, \tag{2}$$

where $P(t)$ represents the measured fluorescence intensity corresponding to the product concentration, $S(t)$ denotes a latent variable corresponding to the substrate, T is the absolute temperature, and B and K_m are constants. To reflect the temperature-dependent catalytic activity of the enzyme, the term $V_{\max}(T)$, which is the maximum rate of product formation, was defined as a function of the absolute temperature T.

The dependence of $V_{\max}(T)$ on temperature was modeled according to the Arrhenius-type relationship by

$$\ln V_{\max}(T) = \alpha\,\frac{1}{T} + \beta, \tag{3}$$

where α and β are constants reflecting the activation energy and the constant factor, respectively.

The product fluorescence exhibited a gradual increase during the first 10 minutes until the droplet temperature was stabilized (see Fig. 3A). A steady nearly linear increase was then observed between 10 and 30 minutes, implying that the reaction rate was saturated. Thus, assuming that the the substrate concentration was much higher than the Michaelis-Menten constant K_m between 10 and 30 minutes, $V_{\max}(T)$ was determined from the slope of the droplet fluorescence intensity.

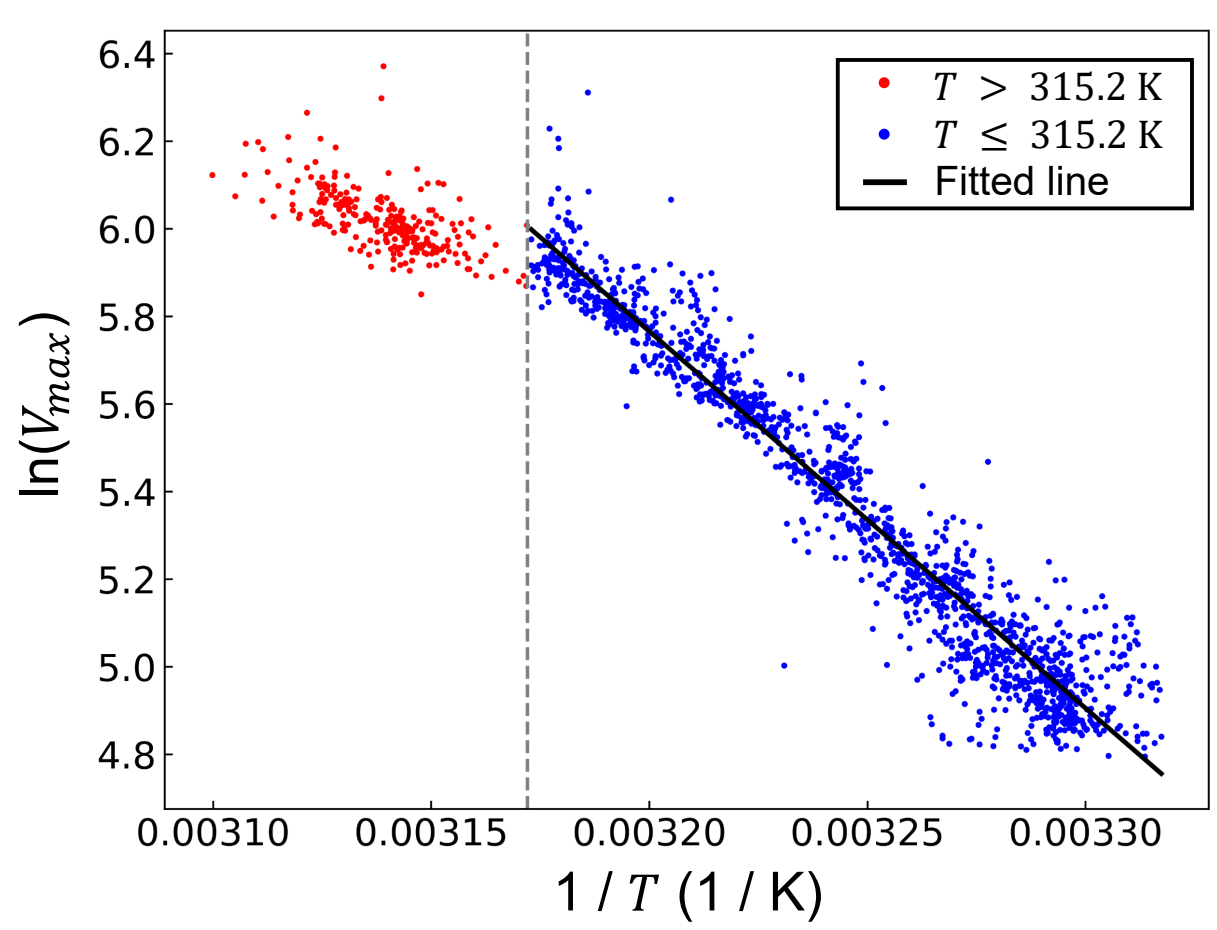

Figure S5: Linear fitting of $\ln(V_{\max})$ with $1/T$ at $t = 30$ min below the transition temperature $T_{\text{break}} = 42.1\,^{\circ}\text{C}$.

Fig. S5 shows the experimentally measured $\ln(V_{\max})$, where $\ln(V_{\max})$ decreases almost linearly with $1/T$ in the low-temperature region around 40 °C (313 K; $1/T = 0.003195$), which is consistent with eq. (3), whereas the slope was not maintained at higher temperatures. This deviation could be due to the faster substrate depletion within the first 10 minutes at high temperatures, where the enzymatic reaction proceeds rapidly and no longer maintains a constant reaction rate. To examine the temperature range over which the linear relationship remains valid, linear fitting was performed for progressively extended temperature windows,

starting from 35 °C (308 K; $1/T = 0.003246$) and increasing the upper bound in 0.1 °C increments. For each temperature window, the mean squared error (MSE) between the observed and fitted $\ln(V_{\max})$ values was computed. The minimum of MSE was achieved for the temperature range below $T_{\text{break}} = 42.1\,°\text{C}$ (315.2 K; $1/T = 0.003173$). Within this windows, linear regression yielded coefficients $\alpha = -8600.09$ and $\beta = 33.29$ (Fig. S5).

The remaining parameters, K_m and B, were determined by fitting the ODE to all time-series data with $T > T_{\text{break}}$. Specifically, the parameters K_m and B were searched over a grid of candidate values ranging from $0.5 \leq K_m \leq 2.5$ (step size 0.5) and $2000 \leq B \leq 4000$ (step size 500). The minimizer of the mean squared error (MSE) between the simulated and observed trajectories was determined as $K_m = 1$ and $B = 2500$ (Fig. S6A).

Finally, the ODE model was evaluated on the test dataset to assess its predictive performance (Fig. 3F). The fitted time-series data corresponding to Fig. 3F is shown in Fig. S6B. Figure S6C shows ODE prediction against all observed data under dynamic temperature gradient, corresponding to Fig. 3F, where only a subset of the time points is displayed. The ODE predictions tend to underestimate the experimental output, indicating that the simple ODE formulation does not fully capture temperature-dependent enzyme dynamics across the entire range.

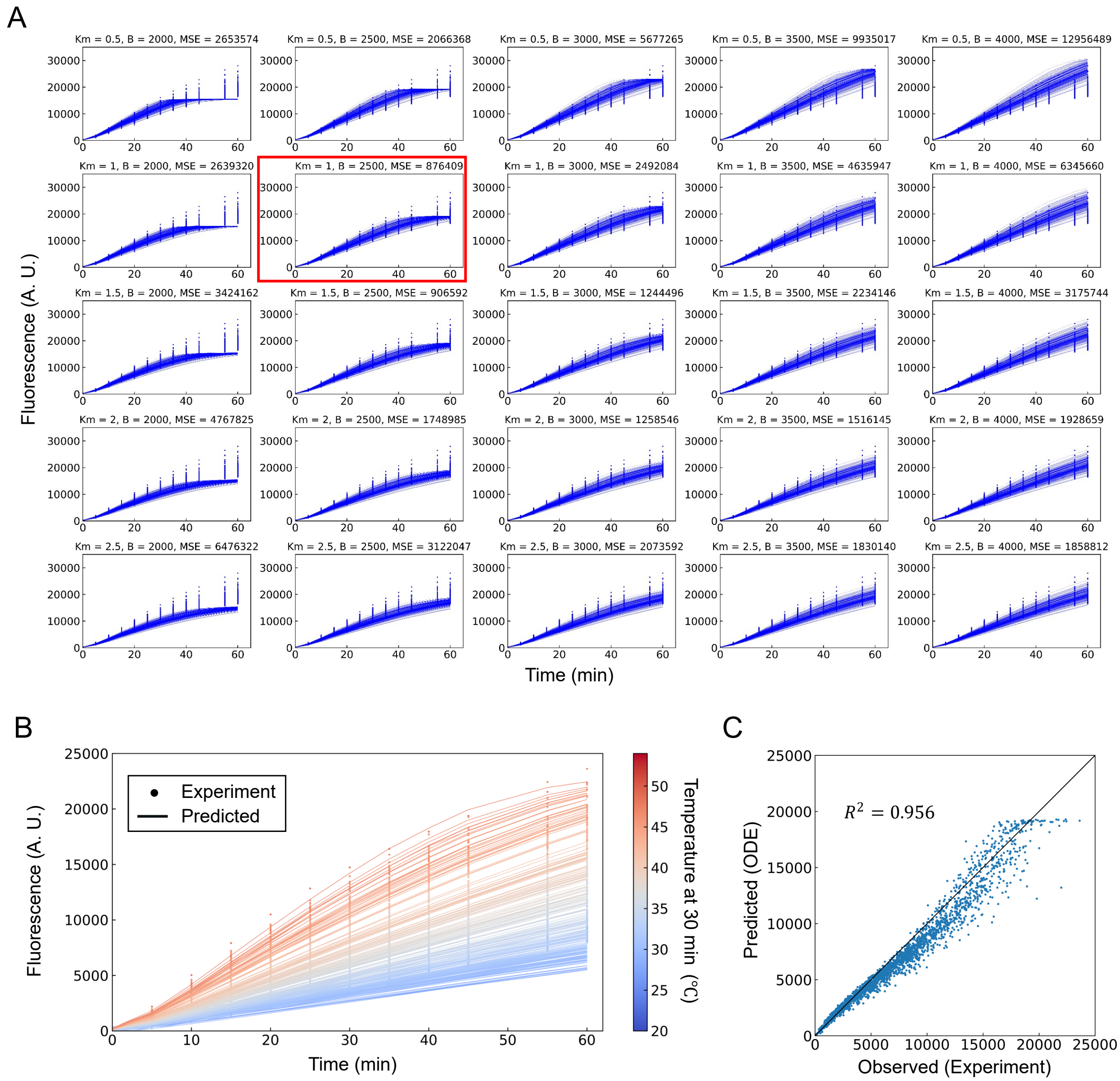

Figure S6: (A) Results of the grid search over candidate values of the parameters K_m and B using training dataset. The parameter combination $K_m = 1$ and $B = 2500$, highlighted by the red box, yields the smallest mean squared error (MSE). (B) Time-series of product fluorescence compared with prediction by ODE model with test dataset. (C) Predicted fluorescence obtained by ODE plotted against experimental data with test dataset.

Supplementary Note 5: Neural ODE model for dynamic temperature gradient

Figure S7 shows the Neural ODE predictions plotted against all test data under the dynamic temperature gradient, corresponding to Fig. 4F in the main text, where only representative time points are displayed. The predicted fluorescence intensities showed a strong linear correlation with the experimental values, indicating that the Neural ODE model successfully reproduced the enzymatic reaction kinetics under dynamically varying temperature conditions.

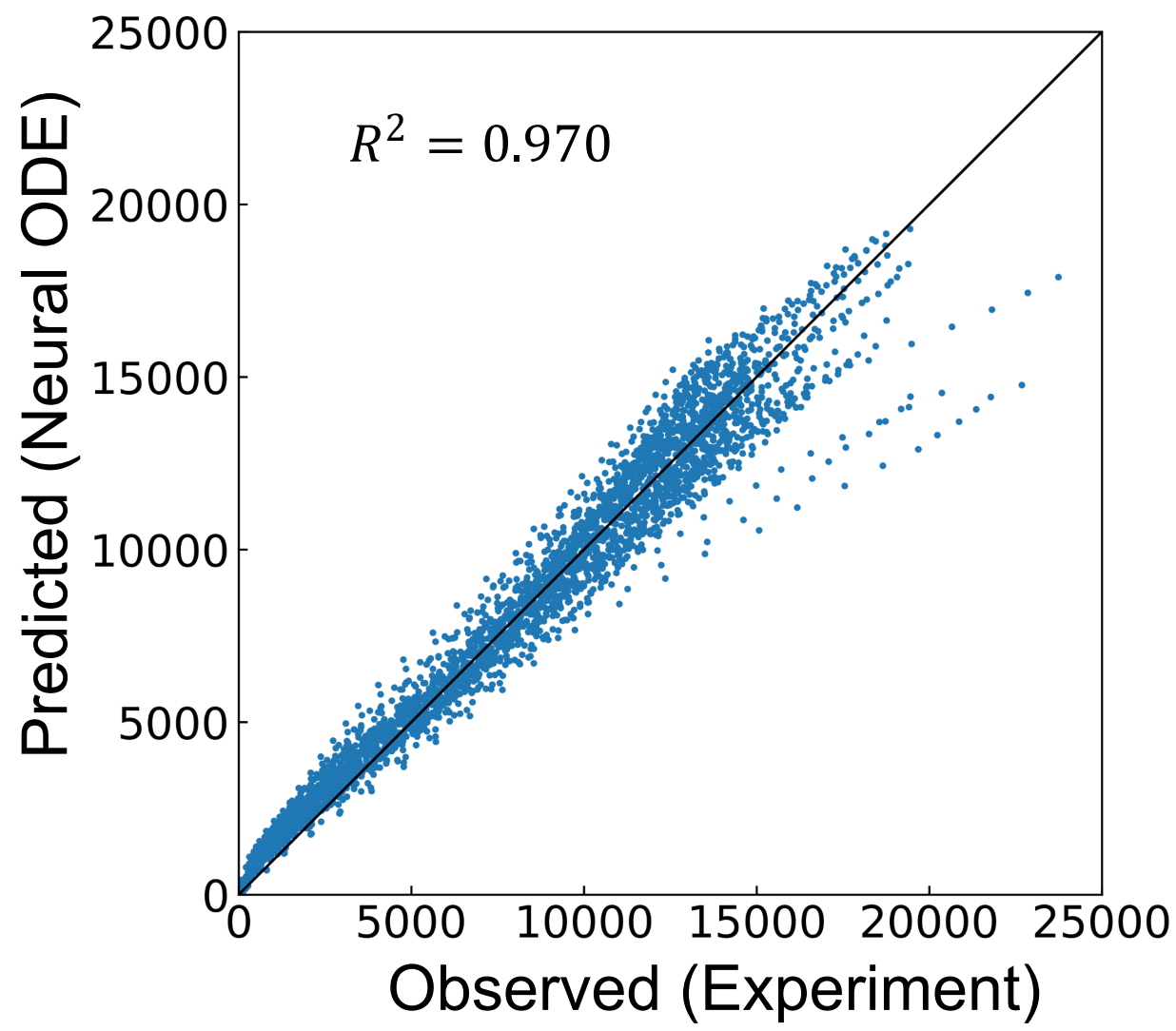

Figure S7: Neural ODE predictions plotted against all test data under the dynamic temperature gradient.

We also performed an architecture sensitivity analysis for the dynamic temperature gradient experiment in Fig. 4. We tested networks with 2, 3, and 4 hidden layers and 64, 128, and 256 neurons per hidden layer. The resulting R^2 scores are summarized in Table S4.

The model with two hidden layers and 64 neurons showed lower predictive accuracy ($R^2 = 0.784$). However, increasing either the number of neurons or the number of hidden layers substantially improved the predictive performance. In particular, the models with two hidden layers and 128 and 256 neurons, and the models with three or more hidden layers achieved high R^2 values of 0.968–0.972. The selected architecture used for Fig. 4, consisting of three hidden layers with 128 neurons per hidden layer, achieved $R^2 = 0.970$, which was comparable to the best-performing architectures while avoiding excessive model complexity. These results suggest that the dynamic temperature gradient experiment requires higher model capacity than the stationary case, but that the prediction becomes robust to the precise architecture once sufficient capacity is reached.

Table S4: Architecture sensitivity analysis of the Neural ODE model under the dynamic temperature gradient. The selected architecture used for Fig. 4 is shown in bold.

Number of hidden layers	Number of neurons	R^2
2	64	0.784
2	128	0.972
2	256	0.970
3	64	0.972
**3**	**128**	**0.970**
3	256	0.972
4	64	0.969
4	128	0.968
4	256	0.972

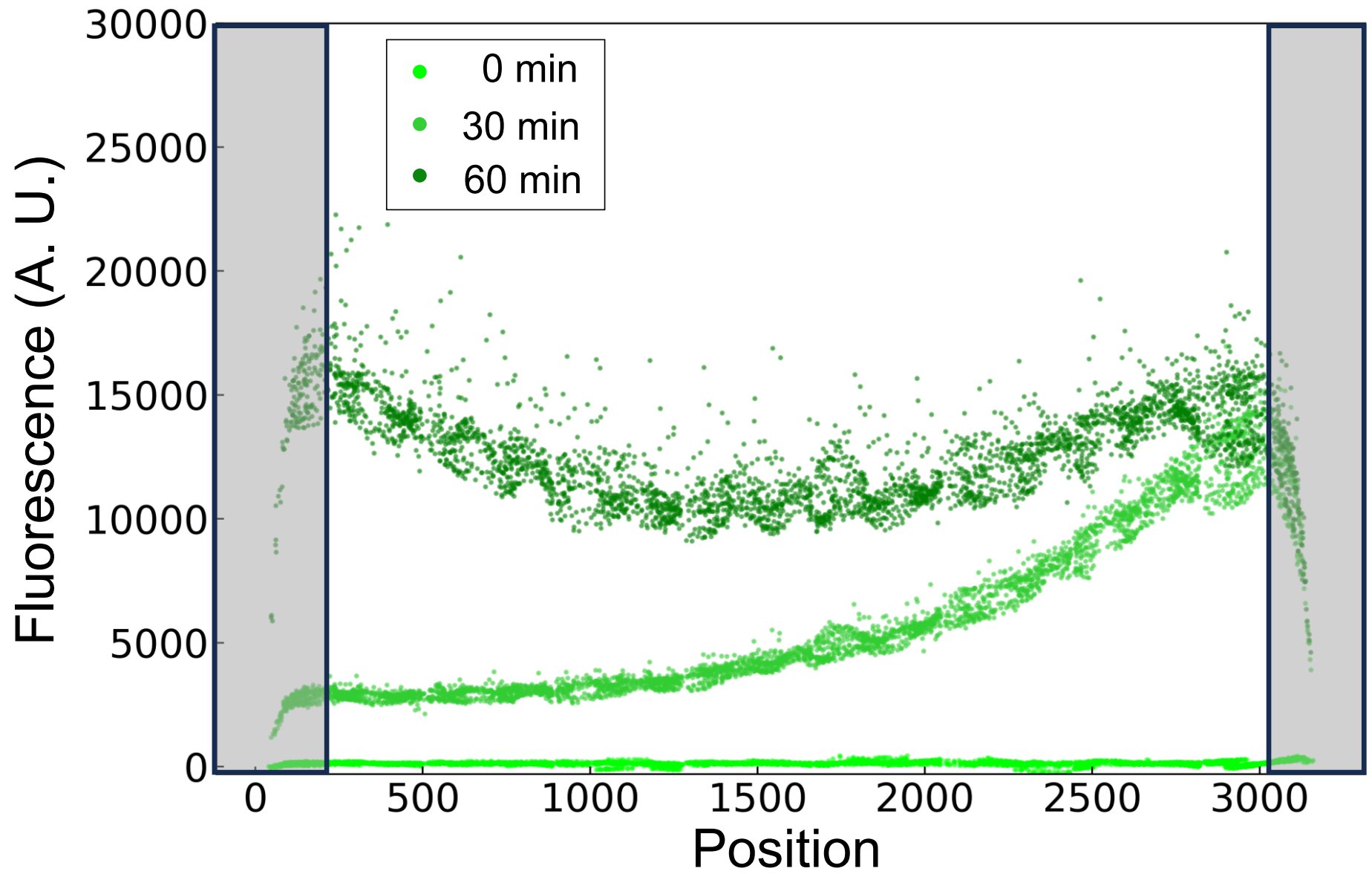

Figure S8: Spatial profiles of fluorescence intensities under the dynamic temperature gradient. The gray hatched regions correspond to the excluded data from the analysis (5% on each lateral side)

Table S5: Sensitivity analysis of the Neural ODE model to the trimming fraction under the dynamic temperature gradient. R^2 stands for the coefficient of determination between the predicted and measured fluorescence intensities on the test set.

Trimming fraction	R^2
0%	0.976
2.5%	0.975
5%	0.975
7.5%	0.976
10%	0.977

Supplementary Note 6: Sequence of DNA Thermometer

The following DNA sequence was used as the DNA thermometer. The strand was labeled with a fluorescent dye (TAMRA) at the 5′ end and a quencher (BHQ2) at the 3′ end. The DNA sequence used in this study was adopted from that reported in literature [2].

Table S6: Sequence of DNA thermometer

Name	Sequence
DNA thermometer	TAMRA-GGGTTTTTTTTTTTTTTT-<br>-TTTTTTTTTTTTTTTTTAACCC-BHQ2

Supplementary Note 7: Temperature control system

The temperature control system used in this study was constructed using two Peltier elements (TEC1-12708, Thermonamic Electronics) and a single-board computer Raspberry Pi (Raspberry Pi® 4 Model B, Raspberry Pi Ltd.) as the main controller. For the purpose of feedback control, temperature was measured with two platinum resistance thermometers (SDT101BXN100DF, KOA), each placed near one end of the glass slide. A schematic overview of the entire temperature control circuit is shown in Fig. S9A. Each platinum resistance thermometer was embedded in a PDMS block as shown in Fig. S9B, and the PDMS block was attached to the surface of the Peltier element (Fig. S9C). A four-terminal sensing method was used to measure the voltage across each platinum resistance thermometer, and the analog voltage signal was digitized using an analog-to-digital converter (ADS122U04IPW, Texas Instruments) before being input to the Raspberry Pi. The temperature was estimated from the measured resistance according to the resistance–temperature characteristics specified in the datasheet of the platinum resistance thermometer.

The temperature of each Peltier element was controlled by applying voltage to the Peltier elements through an H-bridge circuit driven by a motor driver (TB67H450FNG, Toshiba), which enabled both heating and cooling by switching the current direction according to control commands from the Raspberry Pi.

Temperature regulation was achieved by pulse-width modulation (PWM) control implemented on the Raspberry Pi, in which the duty ratio was dynamically adjusted according to the deviation between the target and measured temperatures. The PWM frequency was set to 100 Hz, and the proportional–integral (PI) control was employed to ensure stable convergence to the desired temperature.

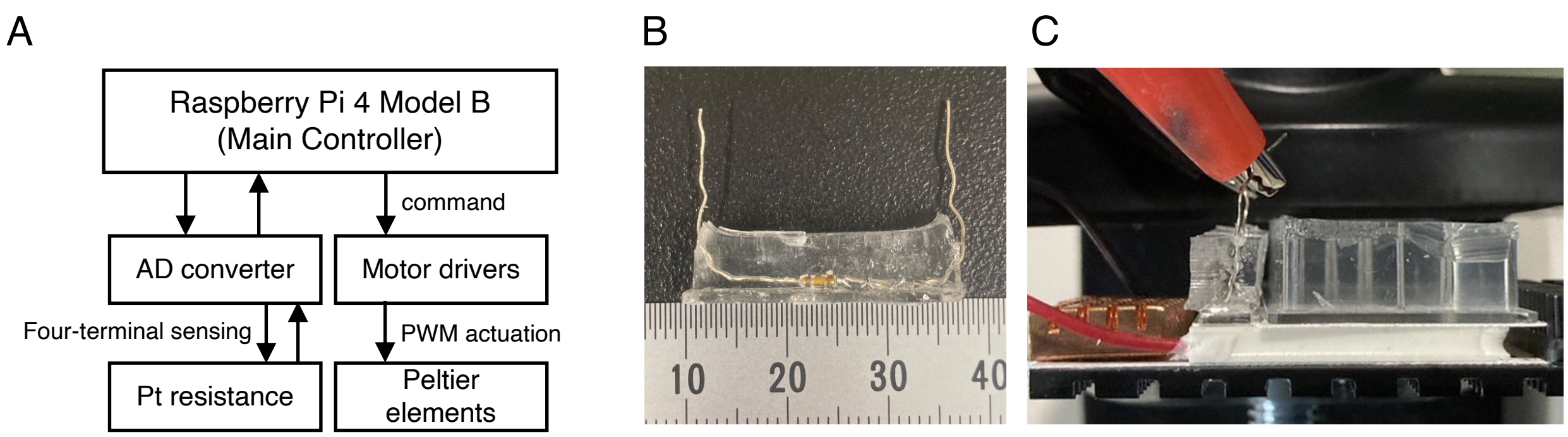

Figure S9: (A) A schematic overview of the entire temperature control circuit. (B) A platinum resistance thermometer encapsulated in a PDMS block. (C) Side view of a platinum resistance thermometer placed on the surface of the Peltier element.

References

[1] Pauli Virtanen, Ralf Gommers, Travis E. Oliphant, Matt Haberland, Tyler Reddy, David Cournapeau, Evgeni Burovski, Pearu Peterson, Warren Weckesser, Jonathan Bright, Stéfan J. van der Walt, Matthew Brett, Joshua Wilson, K. Jarrod Millman, Nikolay Mayorov, Andrew R. J. Nelson, Eric Jones, Robert Kern, Eric Larson, C J Carey, İlhan Polat, Yu Feng, Eric W. Moore, Jake VanderPlas, Denis Laxalde, Josef Perktold, Robert Cimrman, Ian Henriksen, E. A. Quintero, Charles R. Harris, Anne M. Archibald, Antônio H. Ribeiro, Fabian Pedregosa, Paul van Mulbregt, and SciPy 1.0 Contributors. SciPy 1.0: Fundamental Algorithms for Scientific Computing in Python. *Nature Methods*, 17:261–272, 2020.

[2] Noel L. Goddard, Grégoire Bonnet, Oleg Krichevsky, and Albert Libchaber. Sequence dependent rigidity of single stranded DNA. *Physical Review Letter*, 85:2400–2403, 2000.
